# Idiopathic normal pressure hydrocephalus: A sulcal morphometry approach to brain phenotype and clinical response

**DOI:** 10.1016/j.nicl.2025.103816

**Published:** 2025-06-05

**Authors:** Arben Miftari, Fabrizio Pizzagalli, Giulia Bommarito, Stéphane Armand, Frederic Assal, Dimitri Van De Ville, Alessandra Griffa, Gilles Allali

**Affiliations:** a Leenaards Memory Centre, Department of Clinical Neurosciences, Lausanne University Hospital and University of Lausanne, Lausanne, Switzerland; b Department of Neurosciences “Rita Levi Montalcini”, University of Turin, Turin, Italy; c Kinesiology Laboratory, Geneva University Hospitals and University of Geneva, Geneva, Switzerland; d Division of Neurology, Department of Clinical Neurosciences, Faculty of Medicine, Geneva University Hospitals, University of Geneva, Geneva, Switzerland; e Neuro-X Institute, École Polytechnique Fédérale De Lausanne, Geneva, Switzerland; f Department of Radiology and Medical Informatics, University of Geneva, Geneva, Switzerland

## Abstract

Idiopathic normal pressure hydrocephalus (iNPH), the leading cause of reversible dementia in older adults, is characterized by urinary incontinence, gait impairment, cognitive decline, and ventriculomegaly. Neuroradiological features rely on visual assessment, including sulcal characteristics. This study applies automatic sulcal-based morphometry to characterize the sulcal phenotype in iNPH and to distinguish responders from non-responders.

We analyzed the sulcal morphology in 32 iNPH patients and 41 healthy controls. Patients were categorized as responders (Resp) or non-responders (nResp) based on gait improvement following a cerebrospinal fluid tap test. A generalized linear model identified the iNPH sulcal phenotype, and a Support Vector Machine (SVM) classifier was applied to distinguish iNPH patients from controls, as well as Resp from nResp.

We found that sulcal depth and widening were the key descriptors of the iNPH brain phenotype. Eight core sulci contributed the most, including compressed central, superior frontal, and frontal intraparietal bilateral sulci, and flattened left calcarine and posterior lateral fissures. An SVM classifier trained on these features effectively differentiated iNPH patients from controls (AUC: 0.933) but had limited accuracy for Resp vs. nResp (AUC: 0.556). Post-hoc analyses showed smaller superior frontal sulcal opening in nResp than in Resp.

This study identified an iNPH neuroradiological phenotype based on sulcal morphology, emphasizing depth and opening as key markers. SVM classifiers trained on different sulci features performed well in differentiating healthy controls from iNPH patients but was less effective for Resp vs. nResp. Future research should investigate more advanced anatomical landmarks in iNPH.

## Highlights

• Sulcal depth and opening define the structural phenotype of iNPH.
• Eight sulci distinguish iNPH from controls with high SVM accuracy.
• No sulcal phenotype distinguishes responders from nonresponders to the CSF tap test.

## 1 Introduction

Idiopathic normal pressure hydrocephalus (iNPH) is the leading cause of reversible dementia in aging (Allali et al., 2017, Gallia et al., 2006, Williams and Malm, 2016). It is characterized by gait disturbance, cognitive impairment, and urinary incontinence with non-obstructive ventriculomegaly at brain imaging, potentially reversible after surgery. Therefore, identifying appropriate candidates for treatment is critical for patient care. However, iNPH poses a significant diagnostic challenge in clinical practice due to the overlap of clinical symptoms with other neurodegenerative and vascular diseases (Allali et al., 2017, Gallia et al., 2006, Kojoukhova et al., 2015, Williams and Malm, 2016). Because of these diagnostic challenges, a large Swedish study has estimated that only 8 % of iNPH patients receive disease-specific treatment (Halperin et al., 2015).

INPH treatment consists of a cerebrospinal fluid (CSF) shunt procedure. The CSF tap test is a standard predictive test used in clinical practice before considering a shunt procedure (Acosta et al., 2021, Allali et al., 2017, Marmarou et al., 2005, Wikkelsø et al., 1982). It consists of draining an extended amount of CSF via lumbar puncture and observing the patient's response. Gait disturbances are particularly sensitive to changes in CSF dynamics and often exhibit marked improvement following the tap test in responsive individuals (Acosta et al., 2021, Allali et al., 2017). However, the predictive power of the CSF tap test remains variable (Marmarou et al., 2005). Consequently, a negative result does not rule out the possibility of benefiting from treatment, while a positive result does not necessarily confirm the diagnosis (Wikkelsø et al., 2013).

Ventriculomegaly and brain features including cortical volume and thickness alone are insufficient for diagnosing iNPH (Hoza et al., 2015). Besides these features, typical iNPH neuroradiological signs include wide Sylvian fissures, enlarged lateral sulci, and narrowed sulci at the high convexity. This suggests possible disease-specific changes in CSF distribution within the subarachnoid space. These signs are summarized in a semi-quantitative neuroradiological scale (iNPH Radscale) (Kockum et al., 2018) and neuroradiological variant (disproportionately enlarged subarachnoid-space hydrocephalus − DESH) (Shinoda et al., 2017) which contribute to differentiate iNPH from its mimics (Kuchcinski et al., 2019), and responders from non-responders to the CSF tap test (Laticevschi et al., 2021). Yet, semi-quantitative neuroradiological assessments are time-consuming and prone to high inter-rater variability. Consequently, automatic and quantitative assessment of sulcal morphology has been recently used to detect the DESH variant (Gunter et al., 2019) or to distinguish patients with iNPH ventriculomegaly from patients with ventriculomegaly of neurodegenerative origins (Kuchcinski et al., 2019). However, previous studies focused on a few selected sulci and few morphological features, such as the sulcal volume and opening, excluding sulcal depth, length, and area (Kitagaki et al., 1998, Kuchcinski et al., 2019).

Automatic sulcal-based morphometry provides measures of the cortical fissures of the brain and is assessed through brain segmentation (Mangin et al., 2004, Rivière et al., 2002). It has been used as a diagnostic tool for other diseases such as Alzheimer’s disease (Bertoux et al., 2019, Hamelin et al., 2015, Reiner et al., 2012, Mortamais et al., 2022), cerebral small vessel diseases (Jouvent et al., 2008) or schizophrenia (Cachia et al., 2008), and changes in sulcal features and folding patterns are observed in various neurodevelopmental disorders, ranging from cortical dysplasia (Besson et al., 2008) to neurogenetic syndromes (Hong et al., 2000). Sulcal widening has been used as an early sign of atrophy in neurodegenerative conditions (DeTure & Dickson, 2019) as it serves as a sensitive and easily identifiable biomarker of disease progression (Cai et al., 2017, Hamelin et al., 2015).

In this study, we aim to investigate the value of comprehensive sulcal morphology to characterize the iNPH folding phenotype and identify which morphological features contribute the most to the phenotype. Moreover, we assess the discriminative power of these features to distinguish iNPH patients from healthy older adults and their predictive value to CSF tap test response. Establishing the value of sulcal morphology to identify iNPH patients and its predictive value for symptom reversibility can contribute to iNPH diagnostic workflow and clinical management.

## 2 Materials and Methods

### 2.1 Participants and study protocol

**Fig. 1:**
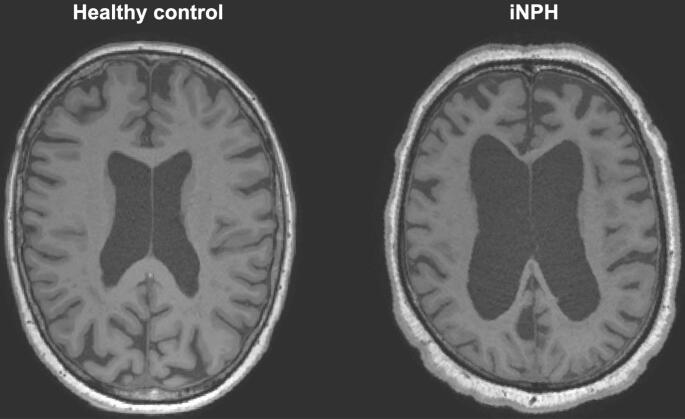
Representative T1w axial MRI images comparing ventricular morphology in a healthy control versus an iNPH patient. The iNPH patient (right) demonstrates significant ventricular enlargement compared to the healthy control (left).

The participants were recruited at Geneva University Hospitals, Geneva, Switzerland, between March 2017 and February 2021, following the Geneva Protocol for iNPH research (Allali et al., 2017). The patients were addressed for suspicion of iNPH to the Department of Neurology of Geneva University Hospitals. The diagnosis of possible iNPH was determined during a consensus case conference with behavioral neurologists and neuropsychologists, following international consensus guidelines (Allali et al., 2017, Relkin et al., 2005). Exclusion criteria were the presence of an acute medical illness in the past 3  months, orthopedic disorders interfering with gait, and a diagnosis of secondary normal pressure hydrocephalus. Healthy control participants were recruited through local advertisements. Eligibility criteria included being over 65 years of age, cognitively normal, free of motor disabilities, and having no contraindications to MRI. Fig. 1 provides an example of a healthy control and an iNPH patient from the examined cohort.

Thirty-two patients (78.9 ± 6.7 years, 14 women) with a diagnosis of possible iNPH and 41 healthy controls (74.9 ± 5.4 years, 30 women) underwent comprehensive neuropsychological and quantitative gait assessments and magnetic resonance imaging (MRI). Patients underwent a CSF tap test the day of and after the assessments, consisting of the removal of 40  ml of CSF with a 20-gauge spinal needle with the patient lying in lateral supine position at the same time of the day. Among the 32 patients, 26 also underwent gait and neuropsychological assessments the day after the CSF tap test and were defined as responders (Resp) or non-responders (nResp). Responders to the tap test were defined as those with an increase of at least 10 % in walking speed and/or a decrease of at least 10 % in the time of the Timed Up and Go (TUG) test 24 h post-CSF tap test following the same criteria from a previous study (Griffa et al., 2022).

This study was approved by the ethical committee of Geneva University Hospitals (protocol NAC11-125), and all subjects provided informed consent according to the Declaration of Helsinki.

### 2.2 Gait assessment

Participants from this study underwent quantitative spatiotemporal gait assessment in a kinesiology laboratory. Subjects walked at their self-selected speed on a 10-meter walkway. Quantitative spatiotemporal gait features were recorded with a 12-camera optoelectronic system (Oqus7+, Qualisys, Sweden) and reflective markers placed on the feet (heel and 2nd toe) to compute average parameters, including walking speed. Additionally, participants performed the TUG test, a validated clinical test assessing mobility and dynamic balance. Its assessment has been used to determine positive response to the CSF tap test (Allali et al., 2017). Both the walking speed and the TUG were acquired before and 24-hour after the CSF tap test. Additionally, we computed the relative change (‘delta’ in Table 1) between the values acquired before (*tp1*) and 24-hour after (*tp2*) the CSF tap test using the following formula: $\mathrm{delta}=\frac{value\left(tp2\right)-value\left(tp1\right)}{\mathrm{value}(tp1)}$.

**Table 1:** Table of demographic, gait, cognitive, and imaging data. The values reported for the continuous variables are the mean and standard deviation. The value reported for categorical variables corresponds to the count of the value in brackets. ‘*tp1*′ means that the measure was acquired before the CSF tap test, whereas ‘*tp2*′ means that the measure was acquired 24 h after the CSF tap test. The ‘*delta*’ between the two time points is computed using the relative difference formula, as follows: $\mathrm{delta}=\frac{value\left(tp2\right)-value\left(tp1\right)}{\mathrm{value}(tp1)}$. This formula is used to determine the delta for the average walking speed, the time to complete the TUG task, and the WAIS cognitive test. P-values are reported for group comparisons between controls and patients and responders (Resp) and non-responders (nResp). Continuous variables were first assessed for normality; when the assumption of normality was not met, the Mann-Whitney *U* test was applied. Categorical variables were assessed with a chi-square test if the sample size was large enough. A Fisher exact test was conducted for data with a limited number of samples per group (e.g., handedness and DESH).

	Patients			Controls		
	All patients	Responders (Resp)	non-Responders (nResp)	All controls	p-values (Controls vs. Patients)	p-values (Resp vs. nResp)
N	32	13	13	41		
age (years)	78.98 ± 6.71	79.84 ± 5.78	79.09 ± 5.24	74.89 ± . 5.36	0.006*	0.83
sex (%females)	43.75 %	61.53 %	61.53 %	73.17 %	0.02*	1.00
handedness (right-handed)	32	13	13	41	1.00	1.00
education level (1/2/3)	17/6/4	5/5/2	9/1/2	5/12/24	< 0.001*	0.14
disease duration (months)	28.96 ± 18.89	26.41 ± 19.65	28.15 ± 15.75	−	−	0.61
WS_tp1 (m/s)	0.72 ± 0.27	0.70 ± 0.28	0.86 ± 0.23	1.22 ± 0.12	< 0.001*	0.17
WS_tp2 (m/s)	0.84 ± 0.29	0.83 ± 0.30	0.85 ± 0.29	−	−	1.00
delta_WS	−	0.22 ± 0.22	−0.025 ± 0.15		−	0.004*
TUG_tp1 (s)	22.29 ± 12.08	23.96 ± 13.50	17.87 ± 8.27	10.69 ± 2.02	< 0.001*	0.18
TUG_tp2 (s)	18.83 ± 10.21	19.65 ± 10.80	18.11 ± 9.96	−	−	0.71
delta_TUG	−	−0.15 ± 0.18	−0.013 ± 0.08		−	0.002*
eTIV_tp1 (cm^3^)	1588.48 ± 136.88	1561.92 ± 80.43	1634.54 ± 153.64	1478.80 ± 145.74	0.002*	0.19
eTIV_tp2 (cm^3^)	1596.53 ± 126.91	1556.75 ± 81.47	1636.32 ± 153.26	−	−	0.21
WAIS_sdm_tp1	28.86 ± 12.12	33.72 ± 8.85	26.67. ± 13.30	54.41 ± 13.36	< 0.001 *	0.13
WAIS_sdm_tp2	32.31 ± 13.02	35.00 ± 12.58	33.55. ± 10.79	−	−	0.93
delta_WAIS	−	0.02 ± 0.13	0.23 ± 0.34	−	−	0.04*
DESH (subjects with DESH)	17	6	7	0	< 0.001*	1.00
Radscale (score)	7.23 ± 2.31	6.61 ± 1.89	7.63 ± 2.38	1.61 ± 1.54	< 0.001*	0.09
FCSRT_ifreecall_tp1	15.04 ± 7.52	16.5 ± 7.39	14.25 ± 8.31	26.2 ± 7.11	< 0.001*	0.50
MMSE_tp1	23.65 ± 1.87	23.90 ± 6.00	23.66 ± 3.57	27.46 ± 1.87	< 0.001*	0.55

Abbreviations: WS: walking speed; TUG: time Up and Go task; eTIV: estimated total intracranial volume; WAIS_sdm: Wechsler Adult Intelligence Scale symbol digit test; FCSRT_ifreecall: Free and Cued Selective Reminding immediate free recall; MMSE: Mini–Mental State Examination.

### 2.3 Cognitive assessment

All participants underwent a comprehensive cognitive assessment, including global cognition and individual cognitive domains. Global cognitive functioning was assessed with the Mini-Mental State Examination (MMSE) (Folstein et al., 1975). Executive functions were probed with the Wechsler Adult Intelligence Scale-III (WAIS-III) symbol digit test (Wechsler, 1997). Memory performances were probed with the French version of the Free and Cued Selective Reminding Test (FCSRT) (Van der Linden et al., 2004). Only the results from the WAIS-III were acquired before and after the CSF tap test, which allows us to compute its relative change using the same formula described above.

### 2.4 MR imaging

All participants underwent an MRI session on a Siemens MAGNETOM Prismafit 3 T scanner equipped with a 64-channel head coil, including a 3D high-resolution T1-weighted (T1w) magnetization-prepared rapid acquisition gradient echo (MPRAGE) sequence with 8 mm isotropic voxel size, 5:03 min acquisition time (TA), 2.4 ms echo time (TE), 2200.0 ms repetition time (TR), 9 deg flip angle (FA) and 230 x 230 x 167 mm field-of-view (FOV). DESH neuroradiological phenotype and iNPH Radscale score (a reproducible semiquantitative grading scale for imaging findings in normal pressure hydrocephalus) were assessed on individual T1w volumes by two experienced neurologists (GA, GB) using the methodologies described in the literature (Kockum et al., 2018, Shinoda et al., 2017).

### 2.5 MR image processing and sulcal morphology assessment

FreeSurfer v6.0.0 (https://surfer.nmr.mgh.harvard.edu/) was used for total intracranial volume (eTIV) assessment and visual inspection. Sulcal extraction and identification were performed using BrainVISA 5.1.2 (http://brainvisa.info). To enhance sulcal extraction, FreeSurfer outputs (orig.mgz, ribbon.mgz, and talairach.auto) were directly integrated into the pipeline. This approach avoids recalculating intensity inhomogeneity corrections and grey/white matter classifications. This protocol is part of the ENIGMA-SULCI working group (https://enigma.ini.usc.edu/protocols/imaging-protocols), and both Docker and Singularity containers have been developed to streamline processing on computational clusters (https://hub.docker.com/repository/docker/fpizzaga/sulci).

Morphologist 2013, an image processing pipeline within BrainVISA, was employed to quantify sulcal measures. This pipeline included computing a brain mask, classifying brain tissue into gray matter, white matter, and CSF, performing gray/white matter surface identification, and a spherical triangulation of the external cortical surface of both hemispheres (Perrot et al., 2011, Rivière et al., 2002, Mangin et al., 2004). Sulci were automatically segmented and labeled according to a predefined anatomical nomenclature comprising 62 sulcal labels for the left hemisphere and 61 sulcal labels for the right hemisphere. From this protocol, we extracted sulcal length, mean width/opening, mean depth, and surface area measurements for 123 sulci. The mean values of opening and depth were used, as they provide more robust measures compared to maximum values and are consistent with methodologies adopted in previous studies (Pizzagalli et al., 2020, Sun et al., 2022). These measures were normalized using z-scores on the whole cohort.

**Fig. 2:**
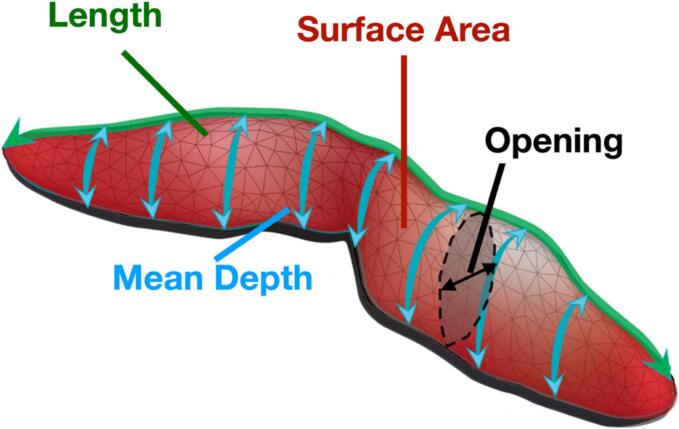
Scheme of the four descriptors computed for each sulcus. The descriptors are color-coded as follows: green represents the length of the sulcus, blue denotes the mean depth, red indicates the total surface area in the native subject’s space, and black corresponds to the mean opening fold, i.e., the width.

Adapted from Sun et al., Nature Communications, 2022, under CC BY 4.0. doi: 10.1038/s41467-022-33829-1. (For interpretation of the references to color in this figure legend, the reader is referred to the web version of this article.)

Fig. 2 illustrates the four measures per sulcus used in this study. For the statistical analyses, only sulci with well-defined values for each metric in each subject (i.e., with no segmentation or labeling errors) were included, resulting in a total of 40 sulci and 160 sulcal measures per subject (Table 2). Previous work highlighted variable segmentation and labeling accuracy levels across sulci, as estimated with the Intraclass Correlation Coefficient (ICC) in multiple test–retest healthy adults’ datasets (Pizzagalli et al., 2020). Based on this previous work, the 40 sulci included in this study’s analyses have high morphology-assessment reliability (ICC = 0.74 ± 0.066) with respect to test–retest data (Pizzagalli et al., 2020).

**Table 2:** Table of the sulci abbreviations and names with their corresponding effect size. The effect size was determined with the standardized beta coefficient from the GLM comparing patients and controls for the four morphological descriptors of the sulci (depth, length, opening, surface). An asterisk following the effect size value indicates that this value is significantly different between patients and controls after correction for multiple comparisons with FDR (alpha < 0.05).

abbreviation	name	Effect size			
		depth	length	opening	surface
F.C.L.p._left	left posterior lateral fissure	−0.753*	0.063	1.104*	−0.493
F.C.L.p._right	right posterior lateral fissure	−0.736*	−0.081	1.069*	−0.650*
F.C.M.ant._right	right calloso-marginal anterior fissure	0.034	0.555	0.376	0.601
F.C.M.post._right	right calloso-marginal posterior fissure	−0.625	0.031	−0.546	−0.370
F.Cal.ant.-Sc.Cal._left	left calcarine fissure	−1.104*	0.272	1.205*	−0.594
F.Cal.ant.-Sc.Cal._right	right calcarine fissure	−0.083	−0.454	0.775*	−0.514
F.Coll._left	left collateral fissure	−0.603	−0.284	0.576	−0.710*
F.Coll._right	right collateral fissure	−0.519	−0.362	0.690*	−0.632
F.I.P.Po.C.inf._left	left superior postcentral intraparietal superior sulcus	−0.811*	0.713*	−0.365	0.255
F.I.P._left	left intraparietal sulcus	−1.276*	0.424	−1.026*	−0.537
F.I.P._right	right intraparietal sulcus	−1.205*	0.176	−1.117*	−0.628
F.P.O._left	left parieto-occipital fissure	−0.651	−0.353	0.364	−0.559
F.P.O._right	right parieto-occipital fissure	−0.330	−0.312	0.410	−0.446
OCCIPITAL_left	left lobe occipital	−0.428	−0.001	0.168	−0.233
OCCIPITAL_right	right lobe occipital	−0.681	−0.280	−0.171	−0.508
S.C._left	left central sulcus	−1.443*	0.349	−1.002*	−0.753*
S.C._right	right central sulcus	−1.165*	0.040	−1.304*	−0.624
S.F.inf._left	left inferior frontal sulcus	−0.269	0.121	0.416	−0.263
S.F.int._left	left internal frontal sulcus	0.193	−0.089	−0.368	−0.052
S.F.int._right	right internal frontal sulcus	−0.286	−0.272	0.001	−0.360
S.F.inter._left	left intermediate frontal sulcus	−0.989*	−0.092	0.346	−0.572
S.F.inter._right	right intermediate frontal sulcus	−0.436	0.290	0.126	−0.001
S.F.marginal._right	right marginal frontal sulcus	−0.563	0.354	0.606*	0.075
S.F.polaire.tr._left	left polar frontal sulcus	−0.510	−0.389	0.069	−0.421
S.F.sup._left	left superior frontal sulcus	−1.374*	0.302	−0.931*	−0.494
S.F.sup._right	right superior frontal sulcus	−1.391*	0.181	−0.503	−0.642
S.Li.post._right	right posterior intra-lingual sulcus	−0.052	0.010	−0.155	−0.088
S.Olf._right	right olfactory sulcus	−0.837*	−0.328	0.838*	−0.750*
S.Or._left	left orbital sulcus	−0.386	−0.102	0.775*	−0.430
S.Or._right	right orbital sulcus	−0.387	0.546	0.696	0.270
S.Pe.C.inter._left	left intermediate precentral sulcus	−0.893*	0.514	0.110	0.157
S.Po.C.sup._left	left superior postcentral sulcus	−0.869*	−0.878*	−0.751*	−1.005*
S.T.i.ant._left	left anterior inferior temporal sulcus	−0.411	−0.136	0.504	−0.233
S.T.i.ant._right	right anterior inferior temporal sulcus	−0.837*	−0.513	0.900*	−0.770*
S.T.i.post._left	left posterior inferior temporal sulcus	−1.073*	−0.283	0.460	−0.646
S.T.i.post._right	right posterior inferior temporal sulcus	−1.088*	−0.039	0.388	−0.638
S.T.pol._left	left polar temporal sulcus	−0.743*	−0.447	0.5846	−0.623
S.T.pol._right	right polar temporal sulcus	−0.613	0.201	0.325	−0.065
S.T.s._left	left superior temporal sulcus	−0.893*	−0.112	0.340	−0.594
S.s.P._left	left sub-parietal sulcus	−0.299	−0.580	0.509	−0.621

### 2.6 Statistical analyses

Initially, the Kolmogorov-Smirnov test was applied to the continuous demographic variables to evaluate the normality of their distribution, revealing that none of the continuous features followed a normal distribution. Then, comparisons between iNPH patients and healthy controls and between Resp and nResp for demographics, gait, cognitive, and semiquantitative imaging data were carried out with a Mann-Whitney U rank test for continuous values (age, cognitive scores, gait scores, and Radscale) and a chi-square test for categorical values (education level, and sex) with enough sample in the different categories. Otherwise, a Fisher exact test was conducted for the other categorical data (handedness and DESH phenotype).

Univariate comparisons of sulcal morphology measures were performed using univariate Generalized Linear Models (GLMs) with age, sex, and eTIV included as covariates, resulting in 160 comparisons (40 sulci × 4 measures). Age and eTIV covariates were normalized using z-scores. The estimated total intracranial volume was included as a covariate to account for interindividual variability in sulcal morphometry associated with head size. Previous research has demonstrated that patients with iNPH exhibit increased intracranial volume compared to healthy controls (Moore et al., 2012, Bradley et al., 2004). Additionally, Krefft et al. (2004) reported larger head sizes in NPH patients relative to controls. These findings support the hypothesis that iNPH may represent a form of congenital hydrocephalus that becomes clinically manifest later in life (Bradley et al., 2004, Krefft et al., 2004). Moreover, previous work investigating sulcal morphology adopted the same strategy to account for global effect (Bertoux et al., 2019; Mortamains et al., 2022; Pizzagalli et al., 2020, Sun et al., 2022).

The effect size was quantified using the standardized beta coefficients of the GLM output. P-values were corrected for multiple comparisons using the False Discovery Rate (FDR) (Benjamini & Hochberg, 1995) controlled at alpha < 0.05. A first GLM was conducted between iNPH patients and healthy controls to highlight an iNPH phenotype, more specifically, which sulci and morphological measures most represent the iNPH phenotype. The same approach was used on Resp and nResp to identify sulcal patterns in these two subpopulations.

A multivariate approach was used to classify participants between groups: controls vs. patients and Resp vs. nResp to the tap test. These analyses utilized linear Support Vector Machine (SVM) classifiers with a Leave-One-Out Cross-Validation (LOOCV) approach to evaluate the classification accuracy. At each iteration, data partitioning was performed where one subject's data served as the test set while the remainder was used for training. Ordinary Least Squares (OLS) regression estimated sulcal measures adjusted for age, sex, and eTIV covariates in the training test. The adjusted metric for the test sample was estimated using the OLS parameters determined on the training set. SVM classifiers were then trained on the adjusted data and evaluated on the adjusted test sample, iterating for each subject. Classification accuracy was quantified with the Area Under the Curve (AUC) of the Receiver Operating Characteristic (ROC) curves. A separate multivariate SVM classifier was trained on the depth and opening of the eight sulci with the largest effect size from the GLM analyses comparing healthy controls and iNPH patients. The number of ‘core’ sulci was arbitrarily selected as the top 20 % of sulcal measures with the largest effect size. Moreover, we focused only on depth and opening because these morphological measures yield the most differences between healthy controls and iNPH patients. Moreover, depth and opening combination was also tested. Similarly, SVM classifiers were trained and evaluated using the top 20 % (n = 8) of sulcal measures exhibiting the largest effect sizes in the GLM analysis comparing Resp and nResp. The SVM models were trained and tested separately on each of the four measures to assess their ability to discriminate between these two groups.

We also investigated the difference in phenotype in the eight sulci highlighted by the GLM analysis between Resp and nResp to the CSF tap test using an ANCOVA with age, sex, and eTIV as covariates. We also considered the controls to highlight if a trend between these three groups is observable. The p-values reported by the ANCOVA were corrected for multiple comparisons using FDR. Moreover, a post-hoc test using Tukey’s HSD test was also conducted to determine if the corrected p-value from the ANCOVA was significant (alpha < 0.05) to highlight groupwise differences.

The iNPH and healthy control groups were not demographically matched; however, analyses were adjusted for relevant covariates, including age and sex.

## 3 Results

### 3.1 Demographic, gait, and cognitive characteristics of the cohort

Demographic characteristics were compared across the study groups, revealing significant differences between controls and patients in terms of age, sex, and education level. Therefore, age and sex were included as covariates in the analyses of the sulci morphology. In contrast, no significant differences were observed between the Resp and nResp groups. Furthermore, several variables, including gait, cognition, and neuroimaging features, were evaluated throughout the study. As shown in Table 1, these three categories of features exhibited significant differences between controls and patients but not between Resp and nResp, especially for eTIV. Indeed, iNPH patients showed a significantly larger eTIV compared to healthy controls (p = 0.002). No significant differences were observed between Resp (N = 13) and nResp (N = 13) patients at any time point (*tp1*: pre-CSF tap test; *tp2*: 24 h post-CSF tap test). When considering changes in gait performance between pre- and post-CSF tap test assessments, we observed a larger improvement in the average walking speed and time to complete the TUG task for the Resp compared to nResp, which was expected given the group definition. The data in Table 1 indicate that Resp tend to exhibit more severe gait impairments compared to nResp at *tp1* (p = 0.17 (walking speed) and p = 0.18 (TUG); however, following the CSF tap test, their performance improved to levels comparable to the non-responder group. In terms of cognitive performance, both groups showed similar results at both timepoints. However, the nResp group shows a significant (p = 0.04) increase in the WAIS symbol digit test score between both timepoints.

### 3.2 Characterization of the iNPH sulcal morphology phenotype

**Fig. 3:**
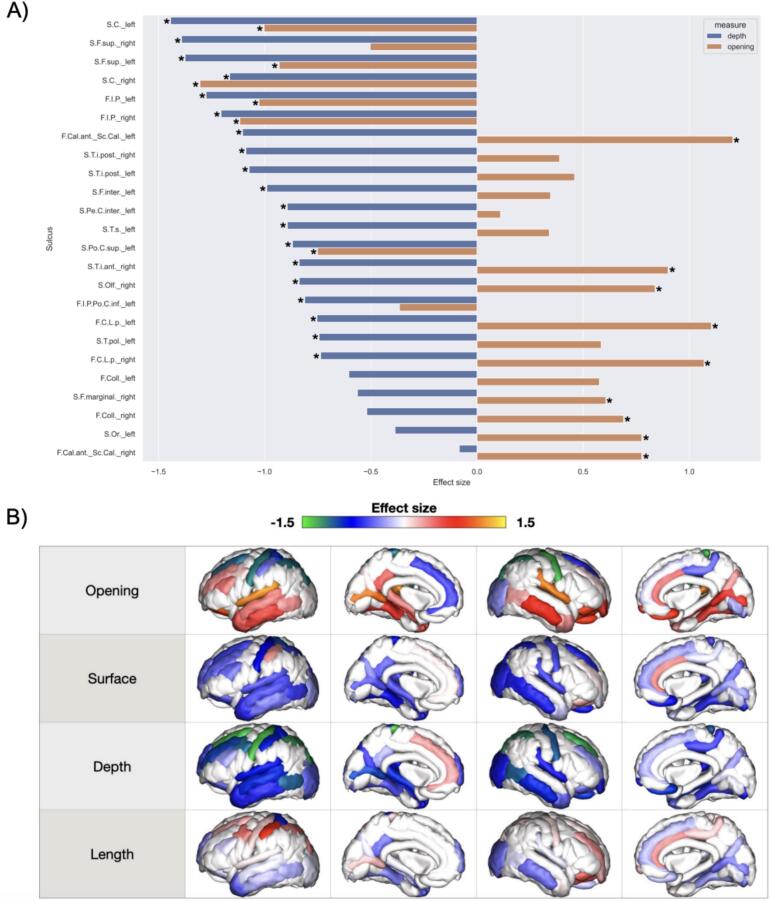
iNPH sulcal morphology phenotype. A: Bar plots of effect sizes for significantly different sulcal depth and opening in iNPH patients compared to healthy controls (HCs). Only sulci with p-value < 0.05 for either depth or opening are included. Asterisks indicate measures that are significantly different between patients and controls after correction for multiple comparisons using FDR. A negative value means that, on average, the corresponding measure is smaller/flattened in patients compared to controls, whereas a positive value corresponds to a larger measure in patients. B: Brain plots of sulcal effect sizes between patients and controls for each sulcal descriptor. Effect sizes are determined using univariate GLM models that include age, sex, and eTIV as covariates and group (iNPH, HCs) as the main effect. Sulci with negative effect sizes (in green and blue) are flattened, compressed, and/or smaller in patients compared to controls, while those with positive effect sizes (in red and orange) are enlarged in patients. The figures, from top to bottom, display effect sizes for sulcal opening, surface area, depth, and length. Sulci abbreviations (left and right sulci are reported separately): S.C. = central; S.F.sup: superior frontal; F.I.P: intraparietal; F.Cal.ant_Sc.Cal: calcarine fissure; S.T.i.post: posterior inferior temporal; S.F.inter: intermediate frontal; S.Pe.C.inter: intermediate precentral; S.T.s: superior temporal; S.Po.C.sup: superior postcentral; S.Olf: olfactory; F.I.P.Po.C.inf: superior postcentral intraparietal superior; F.C.L.p: posterior lateral fissure; S.T.pol: polar temporal; F.Coll: collateral fissure; S.F.marginal: marginal frontal; S.Or: orbital. (For interpretation of the references to color in this figure legend, the reader is referred to the web version of this article.)

First, we aimed to identify which sulci and morphological descriptors better characterize the iNPH sulcal phenotype with respect to the healthy controls. From the univariate GLM analyses, 42 sulcal measures (26 % of the considered measures) were significantly different between patients and controls after correction for multiple comparisons using the FDR (alpha < 0.05). Ranking the effect sizes of the group effect (GLM standardized betas) revealed that sulcal depth and opening are the two descriptors with the greatest number of significantly different sulci between groups (depth: 19 (47 %); length: 2 (5 %); opening: 15 (37 %); surface: 6 (15 %)), and with the associated largest effect sizes. All significant sulci had negative effect sizes for sulcal depth, indicating that sulci were flattened in patients compared to healthy controls, mainly in precentral-postcentral areas (central sulcus and brain vertex), superior-frontal and temporal regions (Fig. 3A, B). Similarly, a negative effect size was observed for the opening of the same precentral-postcentral and superior frontal sulci, indicating sulcal compression in these areas in patients. However, an opposite and positive effect was found in the temporal regions, suggesting flattening and enlargement in these areas (Fig. 3A, B).

To assess the interest in using automatic sulcal morphometry to distinguish patients from healthy controls, a set of linear SVM classifiers was trained on the opening and depth sulcal morphology descriptors and their combination. We focused only on these two descriptors because they contribute the most to the iNPH phenotype, as described above. Only the 20 % of sulci (8 sulci) with the largest effect sizes from the univariate analyses were used for the SVM analyses, namely: the left and right central sulci, the left and right superior frontal sulci, the left and right frontal intraparietal sulci, the left calcarine fissure, and the left posterior lateral fissure. The performances of the classifiers were assessed using a LOOCV approach for each of the two descriptors separately. The results, outlined in Fig. 5, show that sulcal opening (AUC score: 0.894), depth (AUC score: 0.907), and their combination (AUC score: 0.933) are effective measures for distinguishing between patients and controls.

### 3.3 Differences in sulcal morphology between responders and non-responders to the CSF tap test

**Fig. 4:**
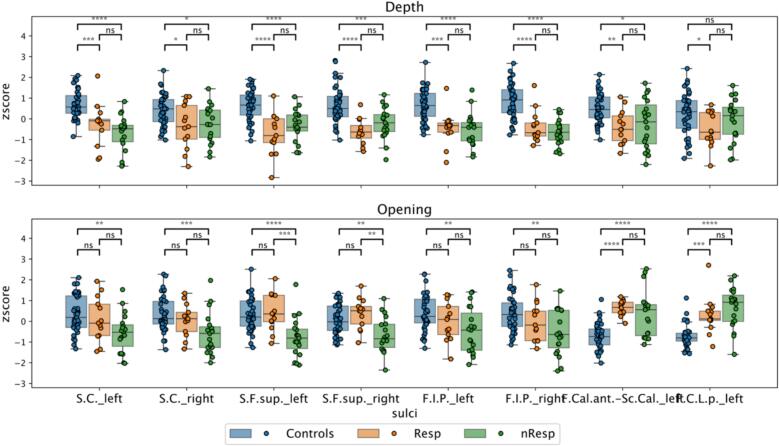
Boxplots of opening and depth descriptors for eight core sulci for healthy controls, iNPH responders (Resp), and non-responders (nResp). The boxplots represent the z-scored morphological values for the depth (top) and opening (bottom) of the left and right central sulcus, the left and right superior frontal cortex, the left and right intraparietal sulcus, the left calcarine fissure, and the left posterior lateral fissure. ANCOVA analyses were used to assess differences among the three groups. If the p-value was below 0.05 after FDR correction for multiple comparisons, post-hoc analysis using Tukey’s HSD test was performed to highlight pairwise differences. Color coding: blue: healthy control group; orange: Resp iNPH group; green: nResp iNPH group. Sulci abbreviations (left and right sulci are reported separately): S.C: central; S.F.sup: superior frontal; F.I.P: intraparietal; F.Cal.ant-Sc.Cal: calcarine fissure; F.C.L.p: posterior lateral fissure. Statistical comparisons: p-value > 0.05: ns (non-significant); 0.01 < p-value < 0.05: *; 0.001 < p-value < 0.01: **; 0.0001 < p-value < 0.001: ***; p-value < 0.0001: ****. (For interpretation of the references to color in this figure legend, the reader is referred to the web version of this article.)

Secondly, we investigated whether the sulcal morphology features characterizing the iNPH phenotype differ between Resp and nResp to the CSF tap test. To this aim, we compared healthy controls, Resp, and nResp, using ANCOVA analyses with age, sex, and eTIV as covariates, and the results are reported in Fig. 4. The ANCOVA p-values were corrected for multiple comparisons (FDR alpha < 0.05). Post-hoc tests assessed groupwise differences using Tukey’s HSD test if the corrected p-value from the ANCOVA test was below 0.05. The post-hoc analysis was performed on the unadjusted means. This analysis was limited to the opening and depth of the 8 sulci most representative of the iNPH phenotype (see previous sections), namely: the left and right central sulcus, left and right superior frontal sulcus, left and right frontal intraparietal sulcus, left calcarine fissure and left posterior lateral fissure.

The distributions of the opening descriptor for specific sulci exhibited a group effect across control subjects, Resp and nResp, especially in the opening of both the right and left central sulci (Fig. 4). Furthermore, significant post-hoc differences were observed between Resp and nResp in the opening of the left and right superior frontal sulci. The distribution of sulcal openings in responders closely resembled that of the control group, whereas non-responders displayed smaller values. An opposite pattern was observed in the left calcarine fissure and left posterior lateral fissure, with the opening in these two sulci being greater in iNPH patients (in both Resp and nResp groups) than in the controls.

Concerning the depth, the distribution of values between Resp and nResp showed no significant differences, as confirmed by the post-hoc analysis. Moreover, control subjects demonstrated significant differences compared to both iNPH groups across most depth measures, as opposed to the opening sulcal measures where the differences between controls and Resp were not significant for most of the sulci measures.

**Fig. 5:**
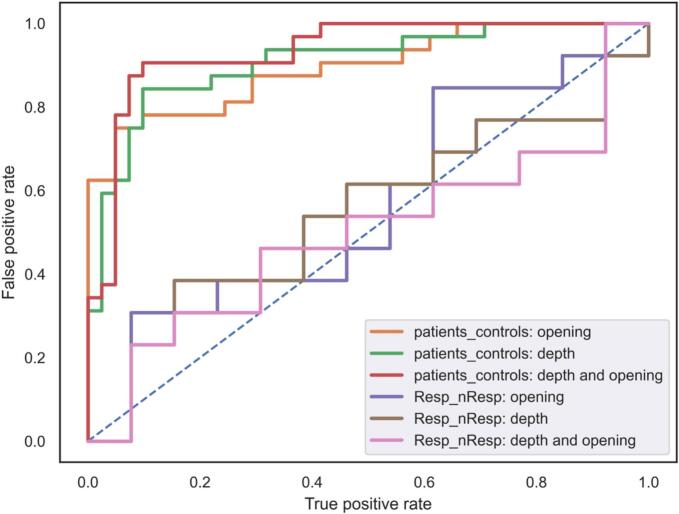
Discriminative power of sulcal morphometry between patients and controls and between responders (Resp) and non-responders (nResp). ROC curves were determined from the performance of the SVM classifiers using a LOOCV approach. Colors indicate the considered groups and sulcal descriptors: orange = patients-controls classification based on sulcal opening (AUC: 0.894); green = patients-controls classification based on sulcal depth (AUC: 0.907); red = patients-controls classification based on sulcal depth and opening (AUC: 0.933); purple = Resp-nResp classification based on sulcal opening (AUC: 0.556); brown = Resp-nResp classification based on sulcal depth (AUC: 0.544); pink = Resp-nResp classification based on sulcal depth and opening (AUC: 0.497). (For interpretation of the references to color in this figure legend, the reader is referred to the web version of this article.)

Using these sulci features, the SVM classifiers were not able to accurately distinguish between Resp and nResp to the CSF tap test. The results exhibited in Fig. 5 show that both depth (AUC score: 0.544) and opening (AUC score: 0.556) produce poorer performance compared to the classifier between patients and controls, and combining depth and opening did not increase the performance of the SVM classifier (AUC score: 0.497). Utilizing sulcal surface area and length for classification did not improve SVM performance, yielding AUC scores of 0.402 and 0.556, respectively.

The GLM analysis comparing Resp and nResp identified eight sulci with the largest effect sizes: right marginal frontal sulcus, left superior frontal sulcus, left polar frontal sulcus, bilateral internal frontal sulci, left sub-parietal sulcus, left central sulcus, and left superior temporal sulcus. SVM classifiers trained and evaluated using these sulci demonstrated poor to moderate discriminative performance: length (AUC score: 0.604), surface area (AUC score: 0.657), sulcal opening (AUC score: 0.651), and sulcal depth (AUC score: 0.479).

## 4 Discussion

The aims of this study were twofold: first, to characterize sulcal morphology in iNPH patients with respect to healthy controls, and second, to train and test a classifier to distinguish between subjects from these groups and iNPH responders from non-responders to a CSF tap test. The results highlighted significant differences between controls and patients and allowed us to identify the most relevant sulcal descriptors that characterize the iNPH phenotype, namely the sulcal opening and depth of the central, superior frontal, intraparietal, calcarine, and lateral fissures. These sulcal measures emerged as suitable features to distinguish between patients and controls when training an SVM classifier. However, they were not able to distinguish between Resp and nResp through the SVM classifier.

Significant differences in population characteristics were observed between controls and patients, especially for the eTIV. It was significantly larger in the iNPH group compared to controls, consistent with previous studies suggesting that iNPH may represent a congenital form of hydrocephalus that becomes clinically manifest later in life (Bradley et al., 2004, Krefft et al., 2004).

Group differences between Reps and nResp groups were noted in the evolution of gait parameters, specifically walking speed and time to complete TUG test, as well as in cognitive performance, measured by the WAIS symbol digit test. Responders demonstrated greater improvement in gait, reaching performance levels comparable to those of non-responders. This may partly explain their positive response to the CSF tap test, as their initially poorer gait function provided more room for measurable improvement. Interestingly attentional performance (WAIS) improved more substantially in the nResp group after CSF tap test. This result was not expected and difficult to explain. However, the presence of comorbid conditions (i.e. Alzheimer's disease) that primarily affect cognition may contribute to this observation.

The results outlined a specific iNPH sulcal morphology phenotype involving a pattern of opening and depth sulcal descriptors. These findings are consistent with a recent study, which reported that the openings of the lateral, superior temporal, and calcarine sulci were significantly larger in shunt-responsive iNPH patients compared to healthy controls and identified the calcarine/cingulate ratio as the most effective parameter for differentiating iNPH patients from healthy controls (Kuchcinski et al., 2019). Our data demonstrated significant differences in the opening of the left and right calcarine fissures between healthy controls and patients. In contrast, for the superior temporal sulcus, significant differences were observed only in the depth measure. Additionally, the sulci around the brain vertex were more compressed compared to the sulci around the lateral part of the brain. This observation is consistent with the DESH phenotype, a neuroimaging variant of iNPH which is defined by enlarged Sylvian fissures, ventriculomegaly, and constricted CSF spaces in the high convexities of the brain (Hashimoto et al., 2010).

We found that the core sulci defining the iNPH phenotype are the left and right central sulci, the left and right superior frontal sulci, the left and right frontal intraparietal sulci, the left calcarine fissure, and the left posterior lateral fissure. The central sulcus being significantly different between patients and controls is particularly interesting. Indeed, the central sulcus exhibits the largest patient-control effect size for both opening and depth descriptors and is located between the precentral and postcentral gyri. The precentral gyrus, where the primary motor cortex is located, plays a crucial role in voluntary motor control (Banker & Tadi, 2023). The postcentral gyrus hosts the primary somatosensory cortex, which is essential for the sense of touch (DiGuiseppi & Tadi, 2024). The proximity of the central sulcus to the primary motor cortex suggests a potential link between its morphology and gait impairment in iNPH, providing possible insights into gait-related dysfunctions. Future research should explore the association between distinct gait phenotypes and the morphology of the central sulcus, focusing on measures such as the sulcal depth and opening or more precise anatomical descriptors like the opening and depth profiles along the sulcus' main geometrical axis (Coulon et al., 2011). A recent study found a correlation between gait phenotypes and cognitive performances (Morel et al., 2024); thus, investigating a similar relationship between gait phenotypes and the central sulcus morphology could yield significant findings.

The superior frontal sulcus is located between the superior frontal gyrus and the middle frontal gyrus in the frontal lobe. Both gyri are involved in higher cognitive functions and specifically in working memory (du Boisgueheneuc et al., 2006, Michalski et al., 2017). Furthermore, they also exhibit significant opening differences between Resp and nResp in post-hoc analyses. Thus, the morphology of these two sulci could help identify responders to the CSF tap test, but further research is needed. The right intraparietal sulcus lies in the parietal lobe and plays a role in higher cognitive functions such as the processing of numerical information (Cantlon et al., 2006), visuospatial working memory (Todd & Marois, 2004), and decision-making (Valdebenito-Oyarzo et al., 2024). It exhibited significant differences between healthy controls and both Resp and nResp iNPH groups. The left calcarine fissure lies in the occipital lobe, which is mainly involved in processing visual information. The left posterior lateral fissure separates the frontal and parietal lobes and is located above the temporal lobe. It corresponds in large part to the Sylvian fissure. The last two sulci are the only ones with a larger opening in iNPH patients (for both Resp and nResp groups) compared to controls. This observation is consistent with the DESH phenotype, which is characterized by enlarged sulci in the lateral parts of the brain, where they both lie. Additionally, the opening of sulci around the vertex, such as the central sulcus and the superior frontal sulcus, is compressed in iNPH patients, which is also consistent with the DESH phenotype. Yet, we note that only half of the iNPH patients in our cohort presented a DESH phenotype (Table 1). Therefore, sulcal morphology differences between patients and controls cannot be explained by the DESH phenotype alone.

The classifier distinguishing iNPH patients from healthy controls showed relatively high performance, indicating that the core sulci features can accurately identify iNPH patients. Implementing an automated system to identify iNPH is beneficial for retrospective studies, which typically rely on expert assessment of medical history and brain imaging features (Kockum et al., 2018) or specific protocols, including gait assessment (Allali et al., 2017). Given that iNPH shares symptoms with other neurological conditions and is often underdiagnosed, a reliable automated classifier could improve identification accuracy despite the presence of comorbidities, which require further research comparing iNPH to mimic disorders (Malm et al., 2013).

SVM classifiers trained and evaluated on the eight sulci with the largest effect sizes from the GLM analysis Resp and nResp failed to effectively differentiate between the two groups. Additionally, the use of the core sulcal features identified in the GLM analysis comparing patients and healthy controls did not enhance classification performance. Further investigation into the depth and opening profiles along the central sulcus' main geometrical axis may uncover more nuanced anatomical distinctions, particularly between responders and non-responders to the CSF tap test. Although the SVM classifier performed poorly, univariate ANCOVA and post-hoc analyses suggest a difference between controls, responders, and non-responders, especially in the central and superior frontal sulci opening (Fig. 4). This observation encourages additional analyses of these sulci using alternative and more advanced anatomical landmarks, which could highlight significant differences that were not detectable with the current measures used. Understanding these morphological changes may also assist in identifying suitable candidates for shunt procedures and provide further insights into gait phenotypes.

This study has limitations. The sample sizes for the different iNPH sub-groups are relatively small (N = 13), which may limit the statistical power and interpretation of the results. Additionally, due to the low number of subjects who underwent the shunt procedure following this study, responders were identified only through the CSF tap test. This method is less accurate compared to determining responders based on the surgical outcome of the shunt. Patients initially included in this study had a diagnosis of possible iNPH with no post-surgical confirmation of diagnosis. A further limitation is the lack of validation in an independent cohort, which may have contributed to the high AUC values observed. Future studies should include external datasets to confirm the generalizability of our findings. The analysis of several imaging features, combined with a relatively small sample size, may further constrain our findings. Moreover, relying on average morphological features such as depth, length, opening, and surface area averaged over the entire sulci may restrict the scope of the analyses.

## 5 Conclusion

This study identified an iNPH phenotype of brain sulcal morphology and demonstrated that sulcal morphology can be used to distinguish iNPH patients from healthy controls. Conversely, iNPH patients responder to a CSF tap test did not exhibit a specific sulcal phenotype with respect to non-responders, although the latter tended to have more compressed superior frontal sulci. Future research could extend these findings by using more advanced anatomical landmarks to highlight significant differences between these two iNPH sub-populations.

## CRediT authorship contribution statement

**Arben Miftari:** Writing – review & editing, Writing – original draft, Formal analysis, Data curation. **Fabrizio Pizzagalli:** Writing – review & editing, Supervision, Software, Methodology, Conceptualization. **Giulia Bommarito:** Writing – review & editing. **Stéphane Armand:** Writing – review & editing. **Frederic Assal:** Writing – review & editing. **Dimitri Van De Ville:** Writing – review & editing. **Alessandra Griffa:** Methodology, Formal analysis. **Gilles Allali:** Writing – review & editing, Supervision, Funding acquisition, Conceptualization.

## Declaration of Competing Interest

The authors declare that they have no known competing financial interests or personal relationships that could have appeared to influence the work reported in this paper.

## Data Availability

Data will be made available on request.
